# Recent developments in fast spectroscopy for plant mineral analysis

**DOI:** 10.3389/fpls.2015.00169

**Published:** 2015-03-24

**Authors:** Marie van Maarschalkerweerd, Søren Husted

**Affiliations:** ^1^Department of Plant and Environmental Sciences, University of CopenhagenFrederiksberg, Denmark; ^2^Foss Analytical A/SHillerød, Denmark

**Keywords:** plant mineral analysis, plant nutrition, nutrient deficiency, UV-Vis spectroscopy, NIR, chlorophyll *a* fluorescence

## Abstract

Ideal fertilizer management to optimize plant productivity and quality is more relevant than ever, as global food demands increase along with the rapidly growing world population. At the same time, sub-optimal or excessive use of fertilizers leads to severe environmental damage in areas of intensive crop production. The approaches of soil and plant mineral analysis are briefly compared and discussed here, and the new techniques using fast spectroscopy that offer cheap, rapid, and easy-to-use analysis of plant nutritional status are reviewed. The majority of these methods use vibrational spectroscopy, such as visual-near infrared and to a lesser extent ultraviolet and mid-infrared spectroscopy. Advantages of and problems with application of these techniques are thoroughly discussed. Spectroscopic techniques considered having major potential for plant mineral analysis, such as chlorophyll *a* fluorescence, X-ray fluorescence, and laser-induced breakdown spectroscopy are also described.

## Introduction

Toward the end of the 20th century, after the Green Revolution, yield growth in agricultural crops slowed down (Gruhn et al., 2000). Now, as an effect of climate changes, local temperature and precipitation patterns are changing, which further challenges yields and farmers’ management practices (Nelson, 2010). With the rapid increase in world population, this puts an immense pressure on food production.

Nutrient management is a major concern for the future, global crop production. At present, over-fertilization causes severe environmental damage, mainly in North America, China and Europe, while especially in the poorest regions of the world, critical depletion of plant nutrients in soils is common (Gruhn et al., 2000; Chen et al., 2008). Wind and water erosion remove the most fertile layers of the soil in many areas, and the amount of nutrient input to the land is generally far from what is removed, causing soil degradation and desertification (Gruhn et al., 2000). In combination with increasing human populations, this lead to a gradual decrease in the worldwide area of cultivated land per person during the period 1961–2009 from a world average of 0.37 to 0.20 ha, a 44% decrease, substantially larger in the poorest countries (WorldBank, 2012). Cultivating marginal and infertile soils is therefore now inevitable in many places, further challenging fertilizer management.

The serious, environmental impact from fertilizers is not limited to the application in the field. Producing especially N fertilizer is highly energy consuming and therefore leads to vast greenhouse gas emissions. In the Western world, natural gas is by far the most common energy source, and for one metric ton of anhydrous ammonia, between 1090 and 1250 m^3^ natural gas is consumed (Gellings and Parmenter, 2004). China, the main consumer of N fertilizer in the world, on the other hand has a production mainly based on coal, often using outdated technologies. By changing to newer production methods, energy consumption of synthesizing NH_4_-N from atmospheric N_2_ could be reduced by more than a third in these cases (Zhang et al., 2013). Due to the high energy demand, prices of energy and inorganic fertilizers were closely related for long (BP, 2012; WorldBank, 2012). However, variations in global demand along with constraints on production capacities in Western Europe and the USA after year 2000 have caused fertilizer prices to stay high even when energy prices were declining (Anonymous, 2005; Schnitkey, 2011; Beckman et al., 2013).

In production of P fertilizer, the main problem is the exploitation of clean rock phosphate, a finite natural resource. The most pessimistic forecasts estimate it to be depleted within 50 years (Gilbert, 2009). A significant geographical concentration, around 75% of global reserves are found in Morocco and Western Sahara (USGS, 2013), may further enhance a global shortage, causing price increases, or fuelling political disagreements. Improving P nutrient use efficiency is therefore crucial, along with the development of new tools to assist plant producers in optimal management of fertilizer application.

Maintenance, and improvement, of soil fertility is a key point in obtaining the most profitable yields. Whilst the marginal soils are increasingly cultivated, the world cannot afford deterioration of fertile lands. To optimize fertilization practices, it is therefore essential for plant producers to assess the nutrient availability in their soil as well as to monitor the performance of crops throughout the growing season. This way it is possible to act in accordance with crop requirements instead of relying on fertilization by tradition or by dubious interpretations of soil analyses, which may not be able to reflect the true plant available concentrations of nutrients. In order to pursue this purpose, accurate, cheaper, and more easily accessible methods for plant and soil mineral analyses are urgently needed.

Presently, a new generation of fast, spectroscopic techniques for plant analysis is emerging. In some cases, nutrient concentrations are assessed, in other cases alternative measures of plant functionality are used as indicators. Common for this group of methods is that they offer instant results with little or no sample handling at a very low cost. However, unfortunately not all methods are properly validated. This paper reviews these new spectroscopic techniques to be used in lab or directly in the field with a critical view on applicability and validity of results.

## Soil Mineral Analysis

**FIGURE 1:**
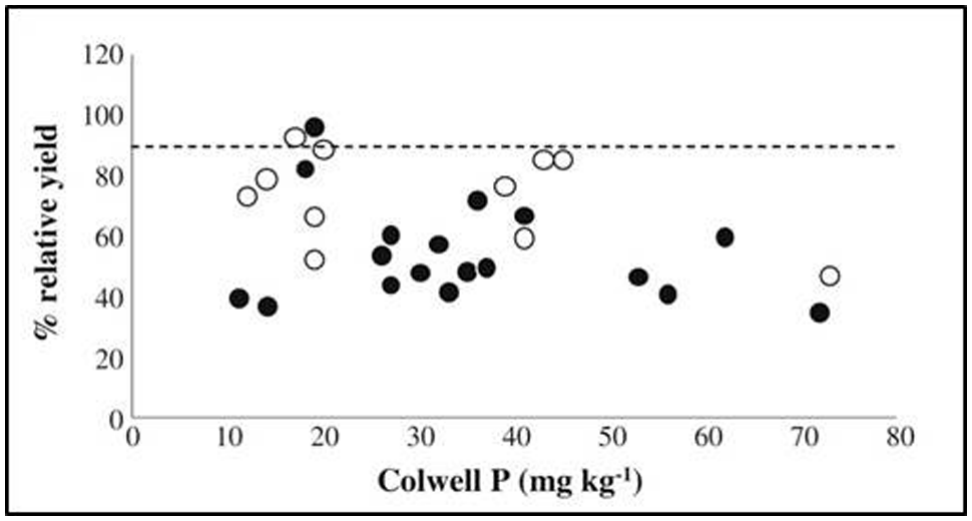
**Relative dry matter yield of wheat at early growth stage (approximately Zadok’s growth stage GS30), plotted against Colwell P extraction results.** From Mason et al. (2010). Relative yields are the observed yields in percent of maximum yields, estimated by fitting a Mitscherlich curve to yields at increasing P rates. Black dots indicate maximum yields were obtained in the experiment, white that they were not.

Presently, and historically, soil chemical testing is widely used in crop production, much more than plant mineral analysis. To demonstrate the proportions, less than 400 plant samples have been registered as analyzed at certified laboratories in Denmark in the growing season of 2012. During the same period, more than 100,000 soil analyses were carried out (Pedersen, 2011, 2012). However, a major disadvantage of traditional soil mineral analysis is the wide array of different methods employed and the time consumption per sample, leading to excessive workloads in laboratories and difficulties in comparing values across countries or regions (Rayment, 1993). Methods for soil analysis are often developed specifically for use in a certain soil type, why combining a range of soil types in an investigation may lead to a complete loss of relation to crop concentrations or yield. This was demonstrated in a meta-study of trace elements, where the correlation between metal concentrations in plant leaves of various species and extracted concentrations in soil was poor or even non-existing (Menzies et al., 2007). For P, a lack of correlation is exemplified in **Figure 1** by plotting early dry matter yield against Colwell P, the most common P extraction method in Australia (Mason et al., 2010). No correlation was found at all between extracted P and early dry matter yield, as the amount of extracted P is highly dependent on soil mineralogy and on the types of chemical bonding between P and soil inorganic and organic fractions. This complicates the use of soil testing for general analysis of fertilizer requirements (Bell et al., 2005; Debnath et al., 2010). In other words, the plant availability of essential plant nutrients may often not be assessed through traditional soil testing methods as they cannot reflect the complex soil chemistry and rhizosphere effects involved.

A new method, DGT, is able to determine plant available concentrations of diffusion-limited nutrients such as P, K, Cu, Zn and the contaminants Pb and As quite well. The background for and application of this method is beyond the scope of the present paper but has been reviewed elsewhere, e.g., Degryse et al. (2009). Also spectroscopy using Vis, NIR, and MIR light (Vis, 400–700 nm; NIR, 700–2500 nm; and MIR, 2500–50,000 nm) has been introduced successfully for determination of several soil characteristics. Light spectra do not contain direct information about atomic concentrations in the soil, why parameters that have been predicted successfully using these methods are mainly physical, in addition to nutrients with strong correlations to spectroscopically active molecules (Malley et al., 1999; Chang et al., 2001; Sorensen and Dalsgaard, 2005; He et al., 2007; Terhoeven-Urselmans et al., 2008; Abdi et al., 2012; Kim and Choi, 2013).

Soil sampling in general has a number of advantages over plant mineral analysis. It is typically, and conveniently, carried out during the less busy seasons when there are no crops in the field, i.e., after harvest or before sowing. This possibility of planning fertilization ahead is practical for the plant producer. Plant mineral analysis on the other hand is more difficult to use, as samples rapidly decay, and data interpretation is more challenging, depending on numerous factors such as plant part, growth stage, species and maybe even cultivar (Lewis et al., 1993; Nabi et al., 2006). Finally, plant mineral analysis has for long not been competitive with soil mineral analysis in terms of price, ease of use, data interpretation and perceived benefits, why soil mineral analysis has remained the dominating tool for fertilizer management in practical crop production. However, the recent, technical developments may very well increase the use of plant analysis.

## Plant Mineral Analysis

The scope of plant mineral analysis differs from that of soil mineral analysis in that it provides a snap shot of the plant nutrient availability up until the day of analysis, and the question of plant availability of nutrients in the soil is, thus, circumvented (Parks et al., 2012). In practice this means that plant mineral analysis today is most frequently used to confirm, or disprove, suspicion of nutrient deficiency related disorders, and in case of poor growth, it will typically be a fundamental part of an investigation of possible reasons. Routine plant mineral analysis used as a guidance tool for fertilization is still less common, but as costs decrease, and the quality and awareness of the advantages increase, the use of plant mineral analysis in practical crop production is likely to intensify.

In Denmark, the use of plant mineral analysis in horticultural production is much more common than in agriculture. One main reason for this is that secondary deficiencies are most prevalent in horticulture. Secondary deficiencies are caused by antagonisms and inadequate translocation of nutrients to plant organs, rather than actual nutrient shortages in the growth medium. Examples are blossom end rot in tomato and bell pepper, and tipburn in Chinese cabbage, which are all Ca related deficiencies. Analyzing for nutrient concentrations in specific plant parts can be of assistance to avoid these disorders. The concentrations of nutrients in plant material in pasture and other forage crops are also commonly analyzed, as these are of major importance to animal nutrition (Römheld, 2012). One barrier to the increased use of plant mineral analysis is the price versus the perceived value by farmers. From the laboratories, much has been done to increase the value of plant mineral analysis, and it is now possible to obtain results in some cases already the same day as the laboratory receives the plant sample. Previously, a processing time of up to 2 weeks could be found, potentially causing major yield losses if any action should have been taken. Prices are still relatively high compared to soil analysis but have declined significantly in recent years. This has caused the use of plant mineral analysis to increase somewhat. Another serious obstacle is the lack of suitable reference material of high quality to correlate the nutrient concentrations to plant health status. Even though comprehensive data collections are found, they do not always represent the plant species and varieties found regionally. This may lead to erroneous conclusions and thereby hamper the success of plant mineral analysis.

**FIGURE 2:**
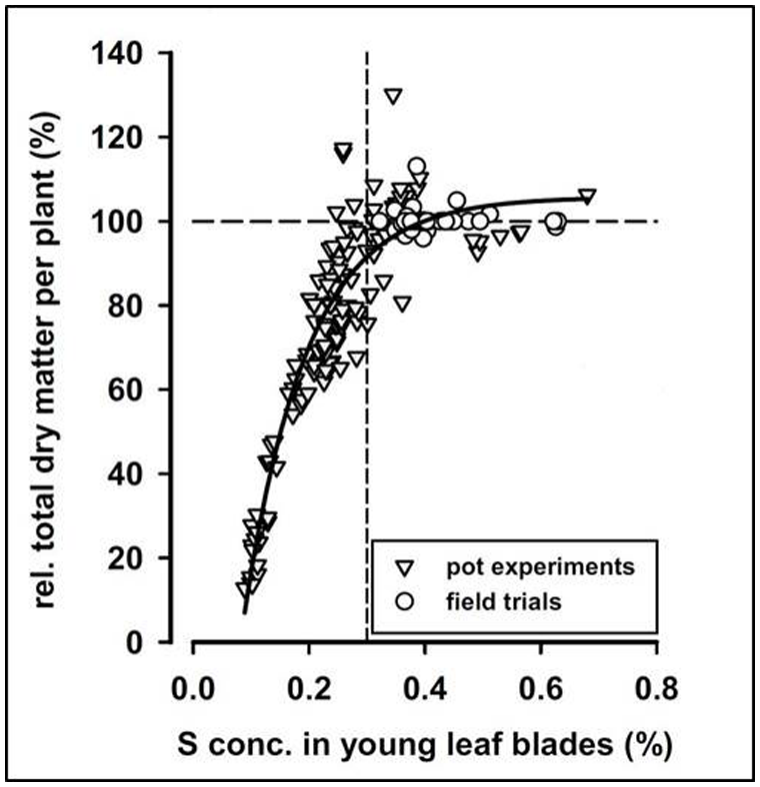
**Relative total dry matter per sugar beet plant at harvest versus S concentration in young leaf blades.** The correlation follows a Mitscherlich curve. From Hoffmann et al. (2004).

The relationship between plant availability, or plant concentration, of a given nutrient and yield or biomass production of the plant is described by the yield-response curve, also commonly known as the Mitscherlich curve (**Figure 2**). The specific shape of the curve may differ, but it generally consists of the same main parts. When an essential nutrient is poorly available, the plant typically develops visual deficiency symptoms on the leaves, enabling diagnosis of the disorder by simple inspection. A less expressed deficiency is called a hidden deficiency, and this can only be discovered with the assistance of advanced analytical methods. If the nutrient is added in excess of the optimum, no additional yield is obtained, and ultimately a yield loss due to toxicity may occur; the latter not shown in **Figure 2**. Providing nutrients in an amount leading to the optimal yield without fertilizing in excess is challenging, and often a slight degree of over-fertilization or hidden nutrient deficiency occurs even in well-managed farmlands.

Deviations from the common Mitscherlich curve occur in specific cases, as for instance the Piper-Steenberg effect (Wikstrom, 1994), and for data close to the maximum yield, alternative functions can be found to represent data better than Mitscherlich. This has been demonstrated, e.g., in Tumusiime et al. (2011). These special cases will not be described here, but the reader is encouraged to refer to the references for further detail.

### Critical Threshold Concentrations

The mineral concentration of one or more nutrients in a specific plant organ is usually compared to a table of CTCs or, more commonly, sufficiency ranges to determine whether the crop is adequately supplied or fertilization is necessary. The CTC is defined as the lowest concentration of a nutrient required for optimal growth and maturation (Ulrich, 1952), and a sufficiency range is simply the concentration range at which plants are adequately supplied. Much effort has been put into defining CTC’s or sufficiency ranges of essential nutrients for most cultivated crops, and the results can be found in large tables as compiled by Reuter et al. (1997). Especially for macronutrients, good correlations are found between plant concentrations and plant nutritional status, typically measured by yield or biomass. In two experiments, S concentrations in corn (Pagani and Echeverria, 2011) and sugar beet (Hoffmann et al., 2004) correlated well to final yields, following a Mitscherlich curve, even though the crops were cultivated on a large variety of soils with different mineralogy (**Figure 2**). It was furthermore possible to determine a CTC for each species.

Factors such as species, genotype, plant age and plant part influence CTC, often substantially (Lewis et al., 1993; Nabi et al., 2006), and general differences in nutrient requirements may be found. For example, dicotyledonous species generally need much higher Ca concentrations than monocots to obtain maximal growth rates (Loneragan et al., 1968; Loneragan and Snowball, 1969). Due to these variations, CTC values need to be specified for each plant species, and it must also be indicated to which plant part and sometimes growth stage, the values apply. In wheat, CTC’s for Zn have been shown to vary by more than 300% between plant parts, from 10.5 mg/kg DW in the stem up to 34.1 mg/kg DW in the ear, making such specifications absolutely essential (Dang et al., 1993). Examples of CTC’s for a selection of common crops are shown in **Table 1**.

**Table 1:** Examples of CTC’s of four common crops (Campbell, 2009).

		CTC (% DW)						CTC (ppm DW)		
Crop	Growth stage	N	P	K	Ca	Mg	S	Fe	Zn	Mn
Corn	Tasseling	3	0.25	2	0.4	0.25	0.12	15	15	15
Wheat	All stages	3	0.15	2	0.15	0.10	0.10	25	15	15
Tomato^a^	All stages	3.5	0.30	3.5	1.0	0.35	0.2	50	18	25
Apple	Mid-season	1.9	0.15	1.25	1.00	0.20	–	50	20	25

*Concentrations apply to youngest fully developed leaf for wheat and tomato, ear-leaf for corn at example growth stage and mid- to terminal leaves for apple.*

*^a^CTC not established. The lowest values of the sufficiency ranges are noted instead.*

**FIGURE 3:**
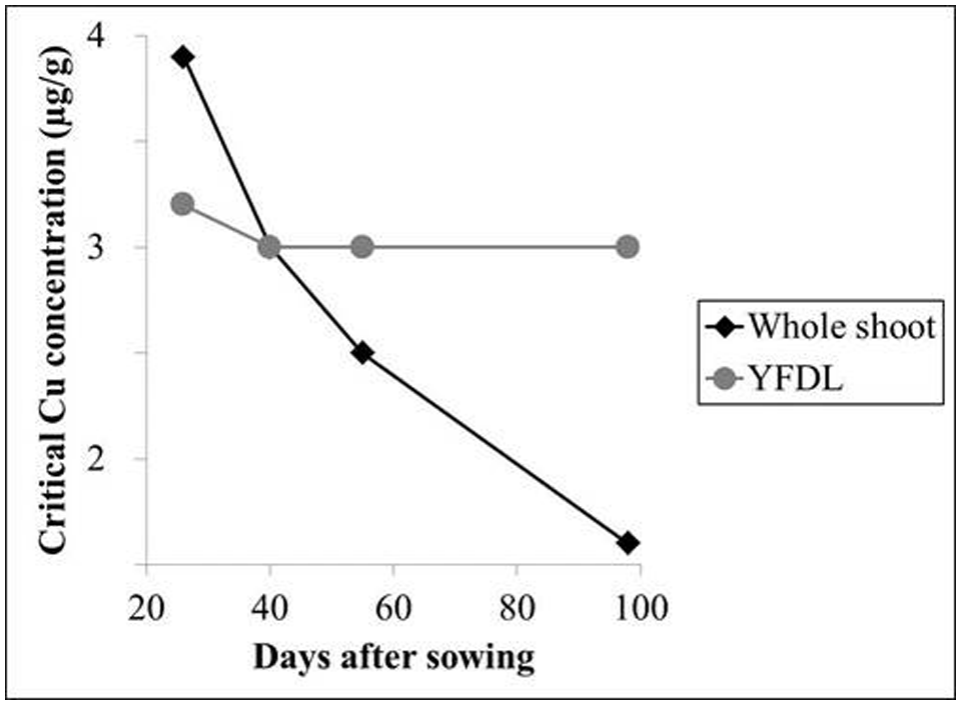
**Critical threshold concentrations for Cu (μg/g) in subterranean clover as a function of plant age.** YFDL, youngest fully developed leaf. Data derived from Reuter et al. (1981).

The physiological age of a plant or plant part affects nutrient concentrations to a considerable degree; after nutrient supply this is the single factor affecting plant nutrient concentrations the most (Römheld, 2012). As plants approach maturity, the nutrient demand for new growth declines, and CTC’s on a whole-plant level decrease for most nutrients, with the phloem immobile nutrients Ca, B, and Mn as the general exceptions (Hill et al., 1979; Römheld, 2012). A dilution effect is a main contributor to this decline. During aging, the plant biomass increases comparably more than the nutrient accumulation, as the proportion of structural (cell walls and lignin) and storage (e.g., starch) compounds grows. This causes a rapid decline of CTC for whole shoots (Römheld, 2012). The youngest leaves on the other hand are only marginally affected by this dilution and therefore have an almost stable CTC throughout the growing period of the shoot, making the youngest fully developed leaf ideal for plant mineral analysis. This important relationship between plant age and CTC levels has been demonstrated for Cu by Reuter et al. (1981; **Figure 3**).

For micronutrients, tissue concentrations are often so low that the use of CTC levels can be very difficult. Contamination of samples and minor errors during digestion and analysis may lead to false conclusions. In a study in wheat and cotton, no significant differences between Cu concentrations in leaves of Cu deficient and sufficient plants could be found (Rao and Ownby, 1993). Supplementing total nutrient analyses with other diagnostic methods when possible can therefore be a great advantage, especially for micronutrients.

### Plant Ionomics

The traditional plant mineral analysis considering only one nutrient at a time has been challenged repeatedly since the late 1980’s (Ingestad, 1987; Parent and Dafir, 1992; Parent et al., 2013). The DRIS is an example of an attempt to include elemental interactions in a diagnosis system using ratios between elemental concentrations and, in its most advanced form, even including interactions with and between soil composition, farming practices and other yield influencing variables (Sumner and Beaufils, 1975). The vast data collection, however, makes DRIS relevant only for high value crops, such as tree fruits, or other perennial cropping systems, e.g., sugar cane, where a crop is cultivated for several years in the same field (Sumner and Beaufils, 1975; Raghupathi et al., 2004; Raj and Rao, 2006; Srivastava and Singh, 2008).

**FIGURE 4:**
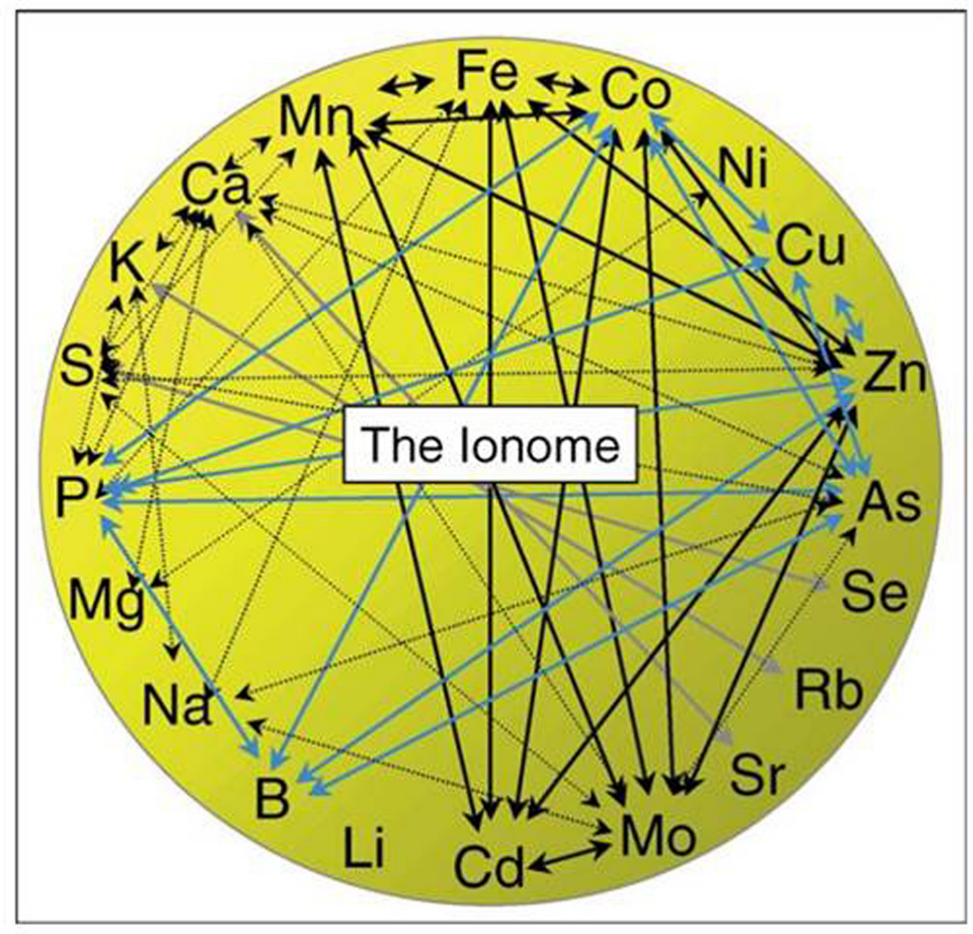
**Genetic, physiological, and chemical interactions between elements; essential, beneficial, and toxic elements are included.** Only a subset of known elemental interactions is presented in this figure. Colors of arrows refer to where the interactions have been described; see reference. From Baxter (2009).

The ionome of a plant or plant part consists of all the elements contained in it, including essential, beneficial and, in some definitions, even toxic elements (Salt et al., 2008; Baxter, 2009). The homeostasis of the plant ionome is controlled by a huge network of interactions between the different elements, a subset of which is presented graphically in **Figure 4** (Baxter, 2009). Experiments that focus exclusively on one or a selected few elements will, thus, be sensitive to alterations in the interactions. With the knowledge of today, it will in most cases not be possible to predict elemental interactions in this giant network, as too many factors of the ionome regulation are still unknown (Baxter, 2009; Singh et al., 2013). Instead, elemental interactions can be determined experimentally; an approach which is becoming more feasible with the developments in multi-elemental analytical methods as well as easily accessible programs for multivariate data analysis.

An overview of the plant ionome will enable diagnosis of physiological and biochemical changes within the plant, and using ionomics, biomarkers for nutrient imbalances as well as other biotic and abiotic stresses may be found. In addition, it may serve as a cheaper and faster method for phenotyping mutants compared to genome sequencing. This is a research area that attracts growing attention, e.g., as seen in reviews by Salt et al. (2008) and Singh et al. (2013).

## Methods of Plant Mineral Analysis

Since the concepts of plant nutrition were founded, much effort has been put into developing methods for diagnosing nutritional disorders. This is usually done by determining the total nutrient concentrations on plants or plant parts, but from using tedious colorimetric or gravimetric methods about a century ago, today much more accurate and swift methods as AAS or the multi-elemental techniques ICP-OES and ICP-MS are employed.

However, alternative, spectroscopic methods based on secondary indices are increasingly gaining foothold. The following is a thorough introduction to these newest methods for fast spectroscopic analysis.

### Fast Spectroscopic Methods

Fast spectroscopic techniques offer rapid plant mineral analysis with instant results. Fast spectroscopy to determine plant nutritional status is a field in rapid development, and several methods are already commercially available and used directly on plants in the field. For assessment of N status of crops, hand-held or even tractor mounted devices with a direct link to fertilizing equipment is being used increasingly (Samborski et al., 2009; Tremblay et al., 2009b; Yara, 2013), and Mn deficiency can be diagnosed directly in the field using a hand-held instrument (Husted et al., 2009; NutriNostica, 2013; Schmidt et al., 2013). At the same time, intensive research is undertaken to improve existing and develop new and better techniques.

Mostly, fast spectroscopic methods require no or only little sample pre-treatment and are therefore often non-destructive. Once the equipment has been acquired, each measurement is essentially free, as no chemicals or disposables are needed. However, measurements are usually indirect, which means that nutrient concentrations are not assessed directly. Instead, compounds (biomarkers) or processes that relate to physiological effects derived from the plant nutritional status are measured, and in the successful cases, these may correlate with plant nutrient concentrations. It is essential that these methods are thoroughly tested to ensure that they provide information specifically about status of the given nutrient. This includes considering if biotic or abiotic stresses may interfere with the results. An insight into the basics of the different spectroscopic methods is given below, in order to provide an understanding of the practical applications.

#### Ultraviolet, Visual, Near- and Mid-Infrared Spectroscopy

Absorption of light in the UV (100–400 nm), Vis, NIR, and MIR parts of the electromagnetic spectrum reflects the molecular composition of a sample and is routinely used for fast analysis in science and industry. Electrons in specific molecular bonds are excited by absorbing the energy from UV and Vis light, and the light absorption of a sample, thus, reflects the concentration of molecules containing these bonds (Herman, 2000). Furthermore, the Vis reflectance indicates the sample color directly.

The basis for NIR and MIR spectroscopy is molecular vibrations. An asymmetric molecule, such as CH_3_-CH_2_-OH or H_2_O, vibrates when exposed to NIR or MIR light, whereas symmetric molecules as Cl_2_ or H_2_C=CH_2_ are not NIR – MIR active. The frequency of molecular vibrations depends on the strength of chemical bonds and the mass of each atom involved. Incoming light with a frequency corresponding to that of the molecular vibrations is absorbed, and the remaining is either reflected or transmitted, thereby yielding information of the sample’s molecular composition (Osborne et al., 1993; Pavia et al., 2001).

In plant mineral analysis, UV, Vis, NIR, and MIR spectroscopy can be used for fast elemental analysis if a consistent correlation between a mineral nutrient and a spectroscopically active compound is present. The mineral may directly form part of the compound or be essential in its biosynthesis. However, any excess of a nutrient will generally not be incorporated into spectroscopically active compounds and therefore not be detectable (Huang et al., 2009). At very low concentrations, a linear relationship between nutrient concentration and spectroscopic data may also fade due to induction of side reactions, and biotic or abiotic stresses can also influence the spectra. The indirect nature of spectroscopic methods for plant mineral analysis therefore makes strict validation of the analytical concentration range and specificity crucial (Zillmann et al., 2006).

#### Chlorophyll Detection by Vis-NIR

A number of commercially available, handheld gages use Vis-NIR for chlorophyll determination in plants (Spectrum Technologies, 2011; Hansatech, 2013; Spinoff, 2013). The SPAD (Soil-Plant Analysis Development) chlorophyll meter, developed already in the 1980’s, measures transmittance at 650 and 940 nm through a leaf and relates the ratio to chlorophyll concentration (Spectrum Technologies, 2011). However, the relation between SPAD readings and chlorophyll concentrations has been shown to be non-linear and differ significantly between species. In addition, it was demonstrated that in some species, the relationship may be very weak (Uddling et al., 2007). Due to the importance of chlorophyll as an indicator of plant health, a continuous development of alternative chlorophyll- and growth parameters does, however, take place.

Tractor-mounted instruments for determination of N status of a crop, based on chlorophyll concentrations, are used today in practical crop production. Two examples are the Yara N-Sensor^®^ and the GreenSeeker^®^. These instruments measure and calculate the NDVI defined as

$$NDVI=\frac{\left(R_{NIR}-R_{red}\right)}{\left(R_{NIR}+R_{red}\right)}$$

Where R_NIR_ and R_red_ designate reflected light at specific wavelengths of NIR and red (620–700 nm) light, respectively. The idea behind the NDVI is that chlorophyll absorbance is high in the red part of the electromagnetic spectrum and low in the beginning of NIR. A ratio between the two gives an approximation of the chlorophyll concentration in the leaf. This is useful in many contexts, but it is not a specific measure of the N status of plants as many other essential nutrients affect the chlorophyll concentration in tissue (Zheng et al., 2009; Römheld, 2012). Using such methods where other factors than N deficiency cause chlorophyll concentrations to decrease may, thus, cause a decrease in N use efficiency with a risk of N leaching and no improvement of yields. However, in fields where N is in fact the growth limiting factor, the distribution of N fertilizer in accordance to chlorophyll concentrations is an effective procedure to optimize yields (Zillmann et al., 2006).

#### Vis-NIR for Nutrient Management

It has been attempted to use Vis-NIR spectroscopy to determine concentrations of most essential plant nutrients in numerous plant species, commonly using chemometrics to relate spectral information to nutrient concentrations. An overview of specific wavelengths and plant materials used in selected papers is provided in **Table 2**. The papers are chosen to present work on a broad range of essential nutrients. Only one paper uses mainly Vis data, combined with the lowest part of NIR (Menesatti et al., 2010), whereas the remaining focus exclusively on NIR or Vis-NIR. It is notable that in the reviewed papers, no investigations have been carried out regarding which exact compounds are reflected in the spectra, though speculations based on spectral inspections combined with theoretical knowledge occur. This aspect will therefore only be sparsely covered in the present review.

**Table 2:** Overview of wavelength ranges and plant materials used in the papers reviewed for Vis-NIR calibrations to determine nutrient status.

Author	Wavelength range (nm)	Plant material	Nutrients
Menesatti et al. (2010)	400–1100	Fresh orange leaves	N, P, K, Ca, Mg, Fe, Mn, Zn
van Maarschalkerweerd et al. (2013)	1000–2500	Fresh barley leaves	Cu
Gonzalez-Martin et al. (2007)	1100–2000	Ground alfalfa	P, K, Ca, Fe, Mn, Zn
Agnew et al. (2004)	400–2500	Dry, ground ryegrass	N
Chen et al. (2002)	400–2500	Dry, ground sugarcane leaves	P
Cozzolino and Moron (2004)	400–2500	Dry, ground lucerne, and clover	S, Fe, Mn, Zn, Cu, B
Dealdana et al. (1995)	1100–2500	Dry, ground grasses	N, P, K, Ca, Mg, Fe, Mn, Zn, Cu
Huang et al. (2009)	400–2500	Dry, ground or cut wheat and rice straw	K, Ca, Mg, Fe
Liao et al. (2012)	1100–2500	Dry, ground tree leaves	N, P, K, Fe, Mn, Zn, Cu
Petisco et al. (2005)	1100–2500	Dry, ground tree leaves	N, P, Ca
Petisco et al. (2008)	1100–2500	Dry, ground tree leaves	K, Mg, Fe, Zn, Cu
Ward et al. (2011)	830–2500	Dry, ground grasses	N, P, K
Villatoro-Pulido et al. (2012)	400–2500	Freeze-dried, ground rocket leaves	K, Ca, Mg, Fe, Mn, Zn, Cu

The indirect correlation between NIR spectra and nutrient concentrations means that great caution needs to be taken during method development and in the use of calibrations. The specificity of a calibration must be ascertained by testing for interference from the most relevant stresses alternative to the nutrient deficiency in question. It is highly remarkable that this is rarely done, though van Maarschalkerweerd et al. (2013) demonstrated that at least for Cu, it is possible to develop a sensitive and specific method that is not influenced by other relevant nutrient deficiencies, based on NIR spectra.

**Table 3** provides an overview of the RPD for data presented in the reviewed papers. The RPD’s are standard deviation of data divided by the RMSEPs or RMSECV, thus, representing the average error on predicted or cross-validated values, respectively. Consequently, the RPD relates calibration performance to the range of measurements and is often used as a quality indicator of the calibration (Ward et al., 2011; Williams, 2014). It does not, however, contain any information about the quality of the validation set, which can be highly variable, from a random subset of the same dataset as the calibration to truly independent samples collected in a different growing season. Furthermore, interpreting the quality of calibrations using RPD values is done using a variety of different approaches (e.g., Chen et al., 2002; Gonzalez-Martin et al., 2007; Huang et al., 2009; Ward et al., 2011). The higher the RPD value, the better the calibration, but which specific value it should surpass to be “good enough” will always depend on the intended use, why such qualitative assertions are not included here.

**Table 3:** Overview of calibration performances in the reviewed papers.

	RPD										
Author	N	P	K	Ca	Mg	S	Fe	Mn	Zn	Cu	B
Menesatti et al. (2010)	2.3	0.7	6.1	1.5	2.0		2.8	3.7	2.7		
van Maarschalkerweerd et al. (2013)										1.4	
Gonzalez-Martin et al. (2007)		2.4	2.3	1.5			2.1	1.4	1.7		
Agnew et al. (2004)	6.5										
Chen et al. (2002)		1.7									
Cozzolino and Moron (2004)						5.6	1.7	1.3	0.6	0.9	1.8
Dealdana et al. (1995)	3.9	1.5	1.8	2.2	1.9		1.8	1.5	1.9	1.8	
Huang et al. (2009), *cut*			1.7	1.8	2.1		1.3				
Huang et al. (2009), *milled*			2.6	2.3	2.6		1.5				
Liao et al. (2012)	2.5	1.4	1.2				1.7	1.5	1.0	1.0	
Petisco et al. (2005)	4.3	2.3		3.8							
Petisco et al. (2008)			2.4		2.2		<3		<3.1	<2.7	
Ward et al. (2011)	1.8	1.4	1.8								
Villatoro-Pulido et al. (2012)			1.9	0.8	1.1		1.2	0.9	1.3	0.9	

*For all relevant elements, RPD is provided. Where RMSEP was not available for the calculation, RPD uses RMSECV instead (Agnew et al., 2004; Gonzalez-Martin et al., 2007; Villatoro-Pulido et al., 2012; van Maarschalkerweerd et al., 2013). Well-performing calibrations are characterized by higher RPD values.*

Versatility of NIR calibrations is often limited, as the composition and distribution of the major, NIR-active components may vary between and within crop types as well as in response to biotic or abiotic stresses (Clark et al., 1987, 1989; Cozzolino and Moron, 2004; Villatoro-Pulido et al., 2012). In one case it was demonstrated that developing common NIR calibrations to predict most macro- and micronutrients for 18 highly different tree species was indeed possible (Petisco et al., 2005, 2008 and **Table 3**), but an attempt to develop common NIR calibrations to determine various nutrient concentrations in rocket leaves (*Eruca vesicaria* subsp. *sativa* and subsp. *vesicaria*) of different genetic origins yielded poor results (Villatoro-Pulido et al., 2012; **Table 3**). This indicates that combining various plant species, geographical origins and growth conditions may be possible in some cases, but a thorough validation is essential before employing a calibration in practice.

Several authors find that calibrations for micronutrients generally perform poorer than calibrations for macronutrients (Petisco et al., 2005, 2008; Huang et al., 2009; Liao et al., 2012). This is supported by **Table 3**, where RPD values above 2 are almost exclusively found for macronutrient calibrations. The reason for the poorer performance of micronutrient calibrations is likely to be the lower tissue concentrations, which lead to a smaller signal to noise ratio and make spectral variation caused by differences in particle size more influential (Yeh et al., 2004; Huang et al., 2009).

In most investigations, NIR spectra are measured on dried, ground plant material. This ensures a homogenous sample and avoids interference from water, which has a very dominant signal in the spectra. The advantage of drying and grinding samples for both macro- and micronutrient calibrations is emphasized by Huang et al. (2009), who demonstrated that RPD values of K, Ca, Mg, and Fe calibrations increased significantly by measuring on dry, milled straw samples instead of cut straw (**Table 3**). In other words, as standard deviations were the same, RMSEP values decreased as a result of drying and milling.

When surpassing nutrient sufficiency levels in plant tissue, the main variation in nutrient concentration is often found in the non-metabolic pool, such as in vacuolar storage and trapping in cell walls, rather than in the pool of metabolically or structurally active nutrients, which could influence the Vis-NIR signal (Lauer et al., 1989; Huang et al., 2009). A significant part of nutrients in well-supplied plants may, thus, stay undiscovered by the Vis-NIR measurements. To develop spectroscopic calibrations for plant nutrient concentrations, it is therefore essential to investigate the valid concentration range and ensure that the majority of calibration samples are found within this range. In a few cases, for P (Ward et al., 2011) and S (Cozzolino and Moron, 2004), a vague tendency of the predictions to approach a constant value above sufficiency concentrations can be noticed, though this is not commented by the authors.

#### Influence of Nutrients on Vis-NIR Spectra

Multivariate calibrations, such as PLS regression, for N concentrations typically make use of the correlation between N and chlorophyll. Further inclusion of the signal from N-H and peptide bonds of proteins indicates a more solid correlation to N concentrations (Dealdana et al., 1995; Petisco et al., 2005). Calibrations for Mg, the central element in chlorophyll, likewise frequently rely on the chlorophyll signal in Vis-NIR calibrations (Dealdana et al., 1995; Tremblay et al., 2009a). However, the proportion of total plant Mg bound in chlorophyll is highly variable. For a fully Mg-supplied plant, as little as below 6% of the Mg content may be bound in chlorophyll. During Mg deficiency this proportion can increase up to 35%, and in combination with low light conditions, which increase chlorophyll concentrations, more than 50% of the total plant Mg may be bound in chlorophyll (Hawkesford et al., 2012). This variability in Mg speciation with chlorophyll weakens the strength of an NIR-based calibration considerably. Numerous factors in addition to Mg and N deficiencies may also affect the chlorophyll concentration, as demonstrated by Ward et al. (2011), who validated their models using data from an independent growing season. This resulted in the lowest RPD in this comparison (**Table 3**) and may be closer to what can be expected if using the method in practice. A comparable increase in error may be expected for Mg calibrations if validated in a similar fashion.

During P deficiency, concentrations of major, NIR-active P fractions such as lipids and esters are highly affected (Petisco et al., 2005; Hawkesford et al., 2012), why NIR-based P calibrations theoretically should perform well. However, the increase in metabolically inactive P_i_ at excessive P concentrations (Hawkesford et al., 2012) might be the reason for the relatively poor performance of P calibrations found in **Table 3**, as compared to other macronutrient calibrations. Further investigating the influence of data range on P calibration performance would be interesting, considering the possible change in effects around the sufficiency threshold.

Qualitative, Vis-NIR-based calibrations for Cu concentrations have not turned out successfully (Dealdana et al., 1995; Cozzolino and Moron, 2004; Petisco et al., 2008; Villatoro-Pulido et al., 2012; van Maarschalkerweerd et al., 2013), and the low concentrations of the nutrient could be a main explanation of this. Instead, it has been demonstrated that Cu sufficient and deficient plants can be clearly distinguished based on NIR spectra already at very early stages of deficiency. The method is specific for Cu deficiency (van Maarschalkerweerd et al., 2013).

The remaining micronutrients investigated, Fe, Mn, Zn and B, also result in calibrations with generally poor prediction power. The only exception is found in Menesatti et al. (2010), where measurements are performed directly on fresh leaves and almost only Vis spectra are included. However, these data represent a very narrow data range, why a comparison to other publications is not feasible.

Visual-near infrared methods carry a large potential for assessment of the nutritional status of crops. Measurements are much faster than traditional laboratory analysis, and using toxic, and expensive, chemicals is avoided. However, there is a lot of work to be done in verifying specificity of methods and assessing concentration ranges and extent of application for each single calibration. Due to the indirect nature of the Vis-NIR nutrient calibrations, this should be subject to continuous investigation during development and application of methods.

#### Fluorescence Spectroscopy and Chlorophyll *a* Fluorescence

Fluorescence is the emission of light during relaxation of excited molecules. Light energy can excite molecules from their ground state, and fluorescence spectroscopy measures the subsequent emission of light when the molecule returns to the ground state. This is a highly sensitive form of spectroscopy, being able to detect very weak signals (Harris, 2007).

Fluorescence measurements are often combined with Vis-NIR. Using multivariate data analysis, this enabled distinction between N sufficient and N deficient potato plants in a greenhouse experiment, even when combined with K and Mg deficiencies (Bélanger et al., 2005). A newly developed, hand-held instrument, Dualex^®^, determines crop N status by measuring the NBI, which is the chlorophyll concentration divided by the concentration of flavonoids. Chlorophylls are determined based on transmittance measurements in the infrared and red ranges, whereas flavonoid concentrations are established by the logarithmic ratio between infrared fluorescence at red and UV excitation light. The NBI ensures a better correlation to N concentration than a simple measurement of chlorophyll, the level of which is affected by a number of factors, as previously discussed (Force-A, 2010; Cerovic et al., 2012; Padilla et al., 2014). However, no investigations have, to the knowledge of the authors, been carried out concerning the influence of alternative nutrient disorders on the NBI.

When light reaches a chlorophyll molecule, one out of three events will occur. The light may be absorbed and used for driving photosynthesis, it can be dissipated as heat or re-emitted as fluorescence, i.e., chlorophyll fluorescence. Only between 2 and 10% of light absorbed by the plant result in chlorophyll fluorescence, but due to the competition between the three processes, chlorophyll fluorescence measurements contain information about the functionality of the photosynthesis (Maxwell and Johnson, 2000; Stirbet and Govindjee, 2011).

**FIGURE 5:**
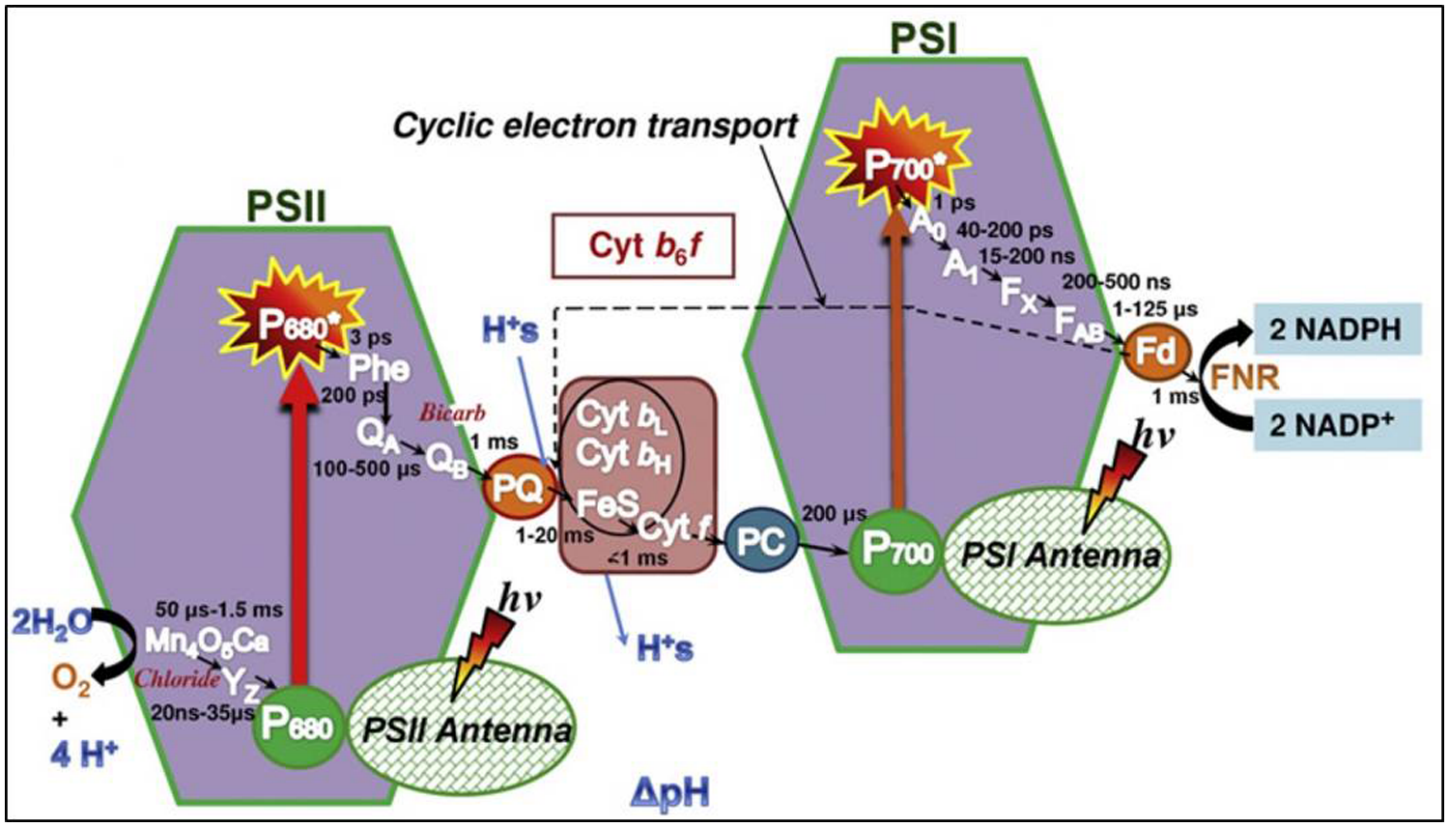
**The Z scheme, illustrating the photosynthetic electron transport chain from PSII via PQ to the cytochrome *b6f* complex.** PC further conveys electrons to PSI, which facilitates the last part of the chain, resulting in reduction of NADP^+^ to NADPH. Figure is from Stirbet and Govindjee (2011).

**Figure 5** shows the so-called Z scheme, which provides an overview of the electron transport chain during photosynthesis. Briefly explained, it shows how electrons are transported from water through the two photosystems via the cytochrome *b_6_f* complex to finally reach nicotinamide adenine dinucleotide phosphate (NADP^+^). Excitation of P680 in PSII provides the energy to transport electrons from water to plastoquinone A (Q_A_), which is then reduced. The Q_A_^-^ delivers the electron to plastoquinone B, then Q_B_^-^, after which the process is repeated to produce Q_B_^2-^. The Q_B_^2-^ then detaches from PSII to join the plastoquinone pool (PQ) as PQH_2_ and delivers the electrons to the cytochrome *b_6_f* complex. From here, electrons are transported to PSI via PC. Finally, on the acceptor side of PSI, electrons are transported to Fd and Fd-NADP reductase, enabling synthesis of NADPH from NADP^+^.

**FIGURE 6:**
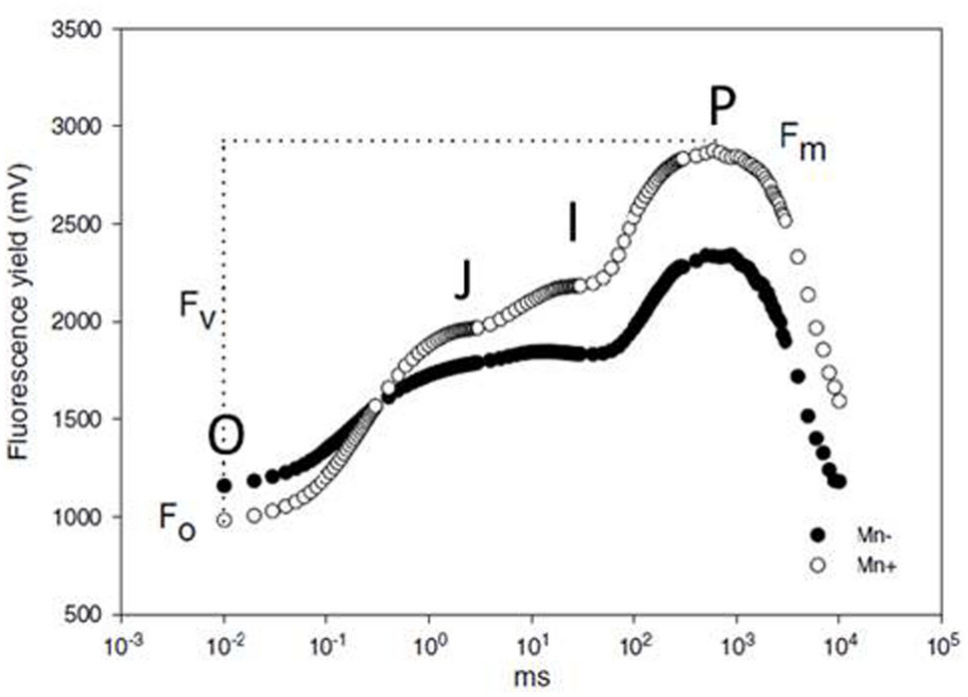
**The OJIP transients of chlorophyll fluorescence for Mn deficient (black circles) and Mn sufficient (open circles) barley plants at the booting growth stage.** From Hebbern et al. (2005).

When a plant is dark adapted, all active PSII reaction centers are open, meaning that all Q_A_ is in the oxidized state. Exposing this plant to a short pulse of actinic light of weak intensity, about 0.1 μmol m^-2^ s^-1^, yields a basic level of chlorophyll fluorescence called the O or F_0_ step (Baker, 2008), which is recognized as the starting point of the fluorescence transient in **Figure 6**. Exposing the same plant to continuous, actinic light at a saturating intensity causes all Q_A_ to be reduced within 1 s, and the intensity of chlorophyll fluorescence forms a curve, commonly known as the OJIP transient or the Kautsky curve. When plotted on a logarithmic time scale, the transient of a healthy plant has four distinct plateaus; O, J, I and P, where P is also called F_m_ (**Figure 6**, open circles). J, I, and P steps are reached at ∼3, 30, and 200 ms, respectively; the exact times may vary slightly among experiments (Schansker et al., 2014).

The first phase of the OJIP transient, from O to J, is called the photochemical phase and is strongly affected by the intensity of the exciting light. The photochemical phase is followed by the thermal phase, from J over I to the P step. The course of this phase is influenced by the temperature during measuring (Schansker et al., 2011; Stirbet and Govindjee, 2011). It has been suggested that the fluorescence in the photochemical phase reflects the electron acceptor side of PSII, or more exactly the first reduction of Q_A_ (Oukarroum et al., 2009; Yusuf et al., 2010; Stirbet and Govindjee, 2011). The J, I, and P steps all seem to represent kinetic bottlenecks in the electron transport chain, and there are indications that they represent electron transport beyond PSII (Schreiber et al., 1989; Schansker et al., 2005). However, the physiological explanations of the OJIP transient, and especially the thermal phase, are still much debated and further knowledge is likely to appear as investigations of the processes continue (Schansker et al., 2014).

Several stresses have been demonstrated to influence the OJIP transient, including salt, drought, and heavy metal toxicity (Oukarroum et al., 2009; Yusuf et al., 2010; Adamski et al., 2011). The relation between plant nutritional status and chlorophyll fluorescence has also been investigated. Light scattering curves during photosynthetic induction, which are roughly the inverts of chlorophyll fluorescence curves (Sivak et al., 1985), of leaves from N, P, K, Mn, Fe, S, or Cu deficient sugar beets were shown to deviate from curves of leaves from healthy control plants by visual inspection. The idea of using such measurements for fast and easy diagnosis of nutritional disorders was presented already in Abadia et al. (1988). However, visible symptoms of the various deficiencies were pronounced at the time of measuring, why these specific results were of little practical use. A fully validated method for fast detection of Mn deficiency in barley has been developed more recently, based on determination of F_v_/F_m_; F_v_ being the difference between F_m_ and F_0_. Rapidly after depriving plants of Mn, the ratio declines below 0.83, the ratio for healthy plants (Hebbern et al., 2005; Husted et al., 2009). **Figure 6** shows examples of chlorophyll fluorescence curves for a healthy and a Mn deficient barley plant. The method has been validated to be specific for Mn deficiency at a time when no visual deficiency symptoms of Mn or other nutrients are present, and other stress factors such as light and temperature can be ruled out. A small, hand-held apparatus has been developed based on this finding, and it is commonly used today, where Mn deficiency is a risk (Schmidt et al., 2013).

#### X-ray Fluorescence

X-ray fluorescence is a spectroscopic technique for multi-elemental characterization of samples, measuring elemental concentrations directly. It exposes sample material to X-rays of appropriate energy to excite the elements in the sample, and during relaxation, X-rays of lower energy are emitted. The energy and intensity of the emitted light is characteristic for each element. For measurements in plant material, quantification of the elements is commonly done by calibrating the XRF instrument against another technique, e.g., ICP-OES. Generally for XRF, the heavier an element is, the easier it is to detect. Thus, heavy trace metals such as Mn, Fe, Cu, Ni, and Zn are easily detectable even in very low concentrations, with limits of quantification down to a few ppm for the heaviest elements. Higher concentrations are needed to quantify S, P, K, Mg, Ca, Cl and Na, whereas B and N are generally not detectable. Measurements are affected by particle size and sample density, and dry leaf material is therefore often ground and pelleted before measuring. For Zn and Fe, concentrations have, however, been determined successfully in smaller grains, such as wheat, rice and pearl millet (Paltridge et al., 2012a,b; West et al., 2012).

Using XRF can be advantageous as a lower-cost alternative to ICP-OES and -MS that is also easier to operate and less sensitive to contamination, if only concentrations of heavier elements are required. Recently, XRF has been used successfully to assess Zn, Fe and Se concentrations in wheat and pearl millet grains in relation to breeding, as well as analyzing the multi-elemental compositions of sunflower and alfalfa under various growing conditions (Gunes et al., 2008, 2009; Paltridge et al., 2012a,b). As XRF measures elemental concentrations directly, different plant species may be combined in a common calibration curve, as shown for P in cotton and corn (McLaren et al., 2012), giving the instrument a high versatility. A major disadvantage of XRF with respect to plant mineral analysis is the time consuming grinding and often pelleting of leaf samples along with the limited applicability for a range of essential plant elements. However, in scientific or agricultural applications where focus may be on a smaller range of elements, the relatively low costs and ease of use makes XRF a promising alternative to atomic spectroscopy in the future.

#### Laser-Induced Breakdown Spectroscopy

Laser-induced breakdown spectroscopy is a technique employing a highly focused laser beam to create a small plasma on a sample surface. The plasma contains excited atomic and ionic species, which emit light as they relax to lower energy states during cooling of the plasma, which lasts only milliseconds. This light is detected and results in a spectrum with specific emission lines for the various species (Cremers and Radziemski, 2006). By use of certified reference material, the detected spectrum is related to total concentrations of elements.

A major advantage of the LIBS technique is the possibility of little or no sample preparation. However, the laser only vaporizes a very small amount of the whole sample, why significant sample inhomogeneity is problematic. This is particularly relevant when working with plant material, where leaves include both veins and leaf blades, which may have very different elemental compositions. Also particle size distribution affects the interaction between laser and sample, why this should be standardized within a group of samples, and physical and chemical properties of the certified reference material used must be comparable to those of the samples (Santos et al., 2012).

Laser induced breakdown spectroscopy is still a new and little explored technique for measuring nutrients in plants. However, with some success, concentrations of K, P, Mg, Ca, Mn, Fe, Zn, and B have been measured in ground and pelleted plant material of wheat, poppy, barley, rape and sugar cane (Pouzar et al., 2009; Nunes et al., 2010).

#### Remote Sensing

The term “remote sensing” is used broadly, from the visual inspection of plants over the tractor mounted sensors described in Section “Chlorophyll Detection by Vis-NIR” and “Vis-NIR for Nutrient Management” and up to the extremes where data is collected from towers, airplanes, satellites, or UAV’s (or simply “drones”). Common methods applied include Vis-NIR, for generation of NDVI, and fluorescence emission. When measuring at very far distances, such as by satellite, the investigations are mainly with the purpose of understanding ecological processes rather than precision agriculture, as the resolution naturally decreases at increasing distances. Nevertheless, Vis-NIR data detected from airborne sensors have been shown to correlate to some extent to grain yield of corn even if affected by N and P deficiencies. The use of UAV’s for precision application of fertilizers and pesticides is rapidly developing, and NDVI has successfully been measured using a UAV. With time and development, the potential of air- and space-borne sensors for large-scale farming may therefore be huge (Osborne et al., 2004; Malenovsky et al., 2009; Hall et al., 2011; Barton, 2012; Bendig et al., 2012; Colomina and Molina, 2014).

#### Perspectives

As the global population continuously grows, harvest yield increases will have to rise accordingly, preferably more, to feed the world. In order to maximize yields on both fertile and marginal soils, optimization of nutrient management is one essential factor. Presently, soil analysis is by far the most used tool to assist plant producers in this, and new methods may improve the usefulness considerably. However, monitoring plant nutritional status throughout the growing season and diagnosing acute disorders depend on accurate plant mineral analysis. With the recent developments toward fast, easy to handle, low-cost methods, it is evident that plant mineral analysis will play a much larger role in fertilizer management in the future than it has done up to now.

New approaches to plant mineral analysis are continuously being tested, as technological advances make them possible. The potato oligo chip initiative (POCI) array is an oligonucleotide potato microarray chip, which can be used to compare gene expression profiles of potato in response to stresses, for instance P deficiency. It is able to determine the expression of 42,034 potato genes simultaneously, which leads the way for multivariate data analysis yielding an overview of changes in gene expression during stresses. A major advantage of the method is the possibility to screen for a wide array of biotic and abiotic stresses at the same time. However, though the POCI has reduced time of analysis significantly, it still takes 2–3 days to obtain results, which in a practical context is relatively long (Kloosterman et al., 2008; Hammond et al., 2011), and the price may be too high for use in practical crop production, where a large number of analyses are needed.

Techniques using fast spectroscopy to determine plant nutritional status still face a number of challenges related to being based on secondary correlations. However, instruments using spectroscopy have proven valuable when used appropriately and are therefore already employed in practical agriculture. Along with the steady development of new techniques for plant mineral analysis, new opportunities arise. Using a single, spectroscopic technique for simultaneous determination of plant status of several nutrients will save time and money, and in addition, superior results are likely to be obtained due to the importance of interactions between elements. As discussed, the interactions and balances between different nutrients may be much more important to plant growth and nutritional health than the mere concentration of each single nutrient and hence contain the key for further understanding of the complexities of plant nutrition.

## Conflict of Interest Statement

The authors declare that the research was conducted in the absence of any commercial or financial relationships that could be construed as a potential conflict of interest.

## ABBREVIATIONS

AAS: atomic absorption spectroscopy
CTC: critical threshold concentration
DGT: diffusive gradients in thin films
DRIS: diagnosis and recommendation integrated system
DW: dry weight
Fd: ferredoxin
ICP: inductively coupled plasma
LIBS: laser-induced breakdown spectroscopy
MIR: mid infrared
MS: mass spectrometry
NBI: nitrogen balance index
NDVI: normalized difference vegetation index
NIR: near infrared
OES: optical emission spectroscopy
PC: plastocyanin
PLS: partial least squares
PQ: plastoquinone
PSI: photosystem I
PSII: photosystem II
RMSECV: root mean squared error of cross-validation
RMSEP: root mean squared error of prediction
RPD: ratio of prediction to deviation
UAV: unmanned airborne vehicle
UV: ultraviolet
Vis: visual
XRF: X-ray fluorescence.
